# A Study on the Energy-Harvesting Device with a Magnetic Spring for Improved Durability in High-Speed Trains

**DOI:** 10.3390/mi12070830

**Published:** 2021-07-16

**Authors:** Jaehoon Kim

**Affiliations:** Department of Advanced Railroad Vehicle Research, Korea Railroad Research Institute, Uiwang 16105, Korea; lapin95@krri.re.kr

**Keywords:** energy harvesting, durability, magnetic spring, generated power, train, wireless sensor

## Abstract

Durability is a critical issue concerning energy-harvesting devices. Despite the energy-harvesting device’s excellent performance, moving components, such as the metal spring, can be damaged during operation. To solve the durability problem of the metal spring in a vibration-energy-harvesting (VEH) device, this study applied a non-contact magnetic spring to a VEH device using the repulsive force of permanent magnets. A laboratory experiment was conducted to determine the potential energy-harvesting power using the magnetic spring VEH device. In addition, the characteristics of the generated power were studied using the magnetic spring VEH device in a high-speed train traveling at 300 km/h. Through the high-speed train experiment, the power generated by both the metal spring VEH device and magnetic spring VEH device was measured, and the performance characteristics required for a power source for wireless sensor nodes in high-speed trains are discussed.

## 1. Introduction

In recent years, the demand for improved reliability and safety of systems and structures has increased owing to accidents and natural disasters, and the increase in maintenance work and cost for these systems and structures has also emerged [1]. Therefore, the development of new technology based on Internet of Things (IoT) sensors and networks is necessary to meet both demands: improving reliability and safety and reducing maintenance work and cost. To achieve this, systems and structures must be intelligent, and the basis of this intelligence is the development of continuous detection technology. To this end, intelligent monitoring with IoT sensors, such as wireless sensor nodes, is required [2].

Recently, technological advancements in IoT sensors, networks, and energy reservoirs (e.g., batteries, supercapacitors) have reduced the power consumption of wireless sensor nodes and have increased the lifetime of intelligent monitoring systems. However, the need for a continuous power supply remains a challenge [3], and an extended power supply for wireless sensor nodes is required for alternative energy sources, such as energy-harvesting technology. The power supply can be increased by utilizing existing environmental energy sources [4,5]. Among the different environmental energy sources, vibration energy exists in various systems and structures and has attracted attention as an energy source that can be harvested [6,7]. Energy harvesting from vibration energy has been applied to wireless sensors, which can be used to monitor the condition of systems and structures, such as bridges [8,9,10,11], pipelines [12], wind turbines [13,14], and vehicles [15,16,17].

The aforementioned technical demands also apply to trains [18,19,20]. The reliability and safety of trains are of great importance, and consequently, maintenance costs have steadily increased. Thus, intelligent monitoring is needed for the early-stage detection of abnormal conditions to prevent failures and accidents [21,22,23]. High-speed trains mostly use wired sensors [24]; however, there are limitations regarding installation and difficulty in modifying the current systems of high-speed trains. Recently, the demand for wireless sensor nodes has increased, as they offer significant benefits. In particular, wireless sensor nodes can be applied to high-speed trains without modifying the systems, thus enabling maintenance based on actual driving conditions while moving. This differs from the current methods used in maintenance management, such as periodic preventive disassembly and inspection. Consequently, the reliability and safety of high-speed trains can be improved [25].

However, although wireless sensor nodes allow for less system modification and fewer location constraints for installation, the power supply problem must be solved for wireless sensor nodes for high-speed trains. Technological advancements in energy reservoirs, such as batteries and supercapacitors, have increased the lifetime of wireless sensor nodes. However, the current energy density rate of batteries does not meet the application demands, and periodic battery replacement is necessary for real-time or long-term wireless sensing of a high-speed train system. Batteries, which consume energy continuously, are a limited resource, are not environmentally friendly, and require additional maintenance [26]. Therefore, wireless sensor nodes require the development of an environmentally friendly and semi-permanent energy-harvesting technology during high-speed train system operation [25,27,28,29,30].

Studies on energy-harvesting devices using electromagnetic induction analyze mechanical motion [27,31,32,33,34], which can be classified into vibrational and rotational motion [21,28,29,35,36,37,38]. An electromagnetic vibration-energy-harvesting (VEH) device is promising for wireless sensor nodes on high-speed trains. A previous study verified the applicability of energy-harvesting technology that produces electrical energy from a vibrational energy source associated with high-speed train operation. An analytical estimation study was conducted on the energy harvested from the high-speed train’s vibration acceleration based on a theoretical model [1]. A design study was also conducted to investigate the potential energy-harvesting power considering the VEH device’s design factors, and a new design for the VEH device was proposed [5]. However, durability is a critical issue in the implementation of a VEH device in a high-speed train. Even with excellent performance, the moving components of a VEH device can be damaged during operation. Studies on the durability of VEH devices were conducted to consider the aspects of design and materials [38,39,40].

In a previous study, design and experimental studies were conducted to determine the amount of energy-harvesting power possible with a design that prevents metal spring damage to improve the durability of a VEH device with a mechanical stopper for use on high-speed trains [41]. A mechanical stopper in the VEH device was the component selected to prevent metal spring damage; however, mechanical stoppers experience hardening during extended use. The increased contact impact of the mechanical stopper and the reduced performance for the limiting displacement of the metal springs in the VEH device (hereafter referred to as a metal spring VEH device) can lead to deformation or fatigue fracture due to stress concentration in the metal spring. Therefore, in this study, in an effort to solve the durability problem of VEH devices, an experiment was conducted to apply a magnetic spring [42,43,44], which is non-contact and not-fatigue damage, to a VEH device using the repulsive force of permanent magnets (hereafter referred to as a magnetic spring VEH device). The experiment was conducted to examine the potential energy-harvesting power using the magnetic spring VEH device in the laboratory. In addition, the characteristics of the generated power were studied using the magnetic spring VEH device in a high-speed train. Through the high-speed train test, the generated power of the metal spring VEH device and magnetic spring VEH device was measured, and the performance characteristics of a power source for wireless sensor nodes in high-speed trains are discussed.

## 2. Durability of Metal Spring VEH Device

**Figure 1:**
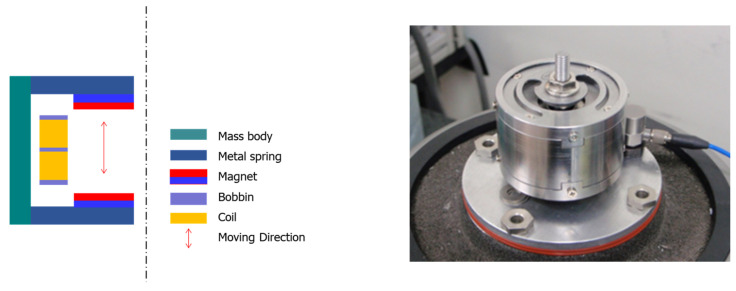
Schematic of the metal spring vibration-energy-harvesting (VEH) device structure (**left**) and the metal spring VEH device for the test (**right**).

A number of experimental studies have been conducted to investigate the energy-harvesting power of VEH devices based on various design factors to verify the applicability of energy-harvesting technology that produces electrical energy from vibrational energy under testbed conditions [16,45,46,47,48]. A previous study verified the applicability of metal spring VEH devices in producing electric energy from a vibration energy source based on the operation of a high-speed train [1,5,41]. The metal spring VEH device was designed so that the same neodymium (Nd) magnets (outer diameter, 25 mm; inner diameter, 10 mm; thickness, 7 mm) were arranged in pairs, and two coils were arranged similarly to correspond to each upper and lower magnet, as illustrated in Figure 1 [5]. The Nd magnet was selected Grade N30AH (the maximum operating temperature of up to 230 degrees Celsius) for the high temperature of VEH device’s inside near the axle box, which is a high-temperature place on the bogie during the high-speed train operation.

**Figure 2:**
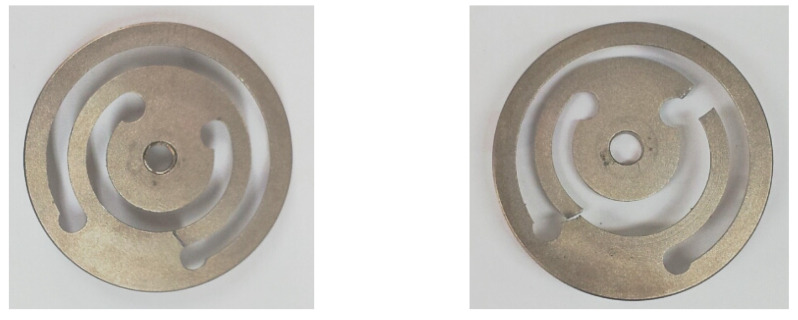
Broken metal springs during high-speed train test [41].

However, durability is one of the main problems in designing a metal spring VEH device. Despite the excellent performance of the metal spring VEH device, the moving components, such as the metal spring, during the operation in a previous study [41,49]. In this case, the metal springs were broken due to the high impact of vibration acceleration during variable operation conditions (velocity, wheel/rail conditions, etc.), as illustrated in Figure 2.

Since metal spring VEH devices use the incoming vibration acceleration load, the vibration acceleration is an energy source and external force that simultaneously influences durability. Therefore, the metal material of the spring is not durable when high vibration acceleration is input in a form such as a periodic impact load.

**Figure 3:**
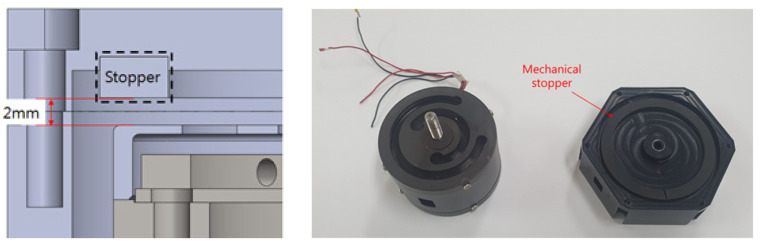
The mechanical stopper of the metal spring VEH device: the maximum moving displacement of the metal spring was 4.0 mm (±2.0 mm) with a mechanical stopper. For the mechanical stopper, a ring-type of ethylene propylene diene monomer rubber material was used.

Therefore, in a previous study, design and experimental studies were conducted to determine the amount of energy-harvesting power possible with a design that prevents spring damage to improve the durability of a metal spring VEH device for use on high-speed trains. As illustrated in Figure 3, a mechanical stopper composed of a ring-type of ethylene propylene diene monomer (EPDM) rubber material (hardness, Shore A65) was the component of the VEH device selected to prevent spring damage [41]. The moving displacement of the spring was 4.0 mm (±2.0 mm) with the mechanical stopper. The generated power (P*_exp-veh_*) is presented in Table 1 and Table 2 for the VEH without and with the mechanical stopper, respectively. The resonance frequency of the metal spring VEH device was 45 Hz. The experimental average generated power (P*_exp_*) was calculated using Equation (1) with the root mean square (RMS) value of the generated voltage (V*_rms_*) and the load resistance (R) [50]:

(1) $$P_{\;exp}=\frac{{\left(V_{rms}\right)}^{2}}{R}$$

However, the EPDM rubber material for the mechanical stopper is subject to hardening over an extended period of time, a characteristic of natural rubber. For this reason, the metal spring has increased contact impact, and the performance of the metal spring is reduced by the limiting performance of the displacement, resulting in stress above the yield strength of the metal spring, which leads to deformation or fatigue failure of the metal spring. Therefore, to solve the durability problem of the metal spring VEH device, this study investigated the use of a magnetic spring VEH device.

## 3. Design of Magnetic Spring VEH Device

### 3.1. Laboratory Experiments to Examine Characteristics of Magnetic Spring Using Magnetic Repulsive Force Tester

**Figure 4:**
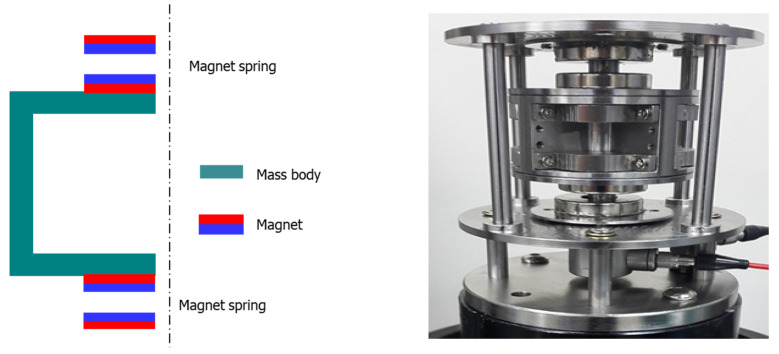
Schematic of the magnetic spring (**left**) and the magnetic repulsive force tester with the load cell (**right**).

A schematic of the magnetic spring is presented in the left part of Figure 4. The resonance frequency of the magnetic spring VEH device is based on the weight of the mass body and the repulsive force of the magnetic spring, and the spacing between the two magnets determines the repulsive force of the magnetic spring. Therefore, in the laboratory experiment, the repulsive force of the magnetic spring was measured according to the spacing between the magnets. In addition, the maximum force, which was regarded as the maximum dynamic load, and the resonance frequency were measured according to changes in the weight of the mass body for the design of the magnetic spring VEH device applicable to high-speed trains.

**Figure 5:**
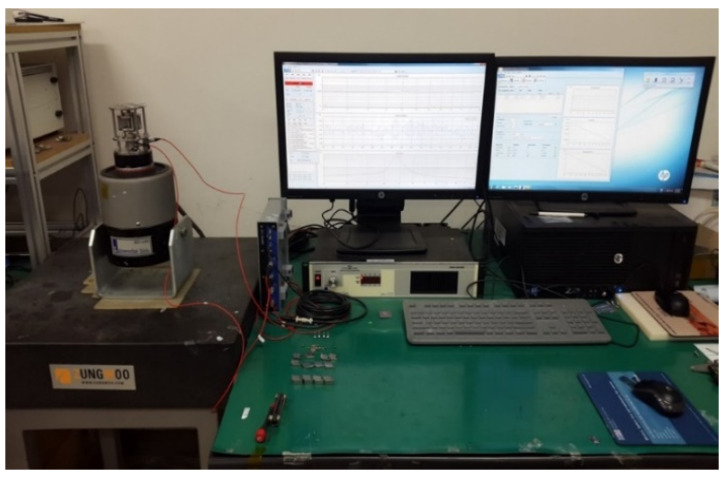
Measurement equipment and magnetic repulsive force tester on a vibrator for measuring the maximum force and resonance frequency.

As illustrated in Figure 4 and Figure 5, the repulsive force of the magnetic spring was measured using a magnetic repulsive force tester, which was constructed in this study, for the preliminary design study of the magnetic spring VEH device. The magnets in the magnetic repulsive force tester were the same and had the same size (outer diameter, 25 mm; inner diameter, 10 mm; thickness, 7 mm) as the ring-type permanent Nd magnet used in the generating core of the metal spring VEH device. This was for ease of design and manufacture of the VEH device. The magnetic spring was composed of a pair of Nd magnets at the top and bottom of the magnetic repulsive force tester. A mass body corresponding to the generating core of the VEH device was added between the magnetic springs to simulate the same conditions as in the VEH device. The mass body was designed to be detachable so that its weight could be varied. In addition, the spacing between the magnets was varied by a plate, which was on the upper and lower sides of the magnetic repulsive force tester, to adjust the magnet up and down. A load cell was on the bottom of the lower plate of the magnetic spring to measure the change in the maximum force corresponding to the maximum dynamic load under the test conditions.

**Figure 6:**
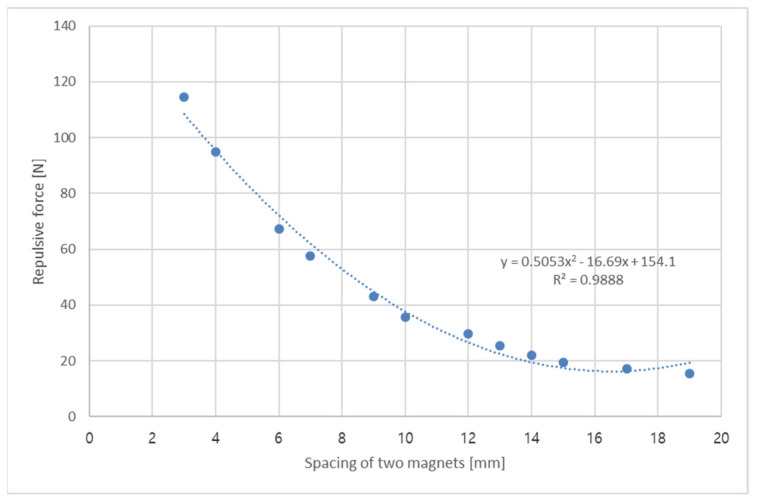
Repulsive force of the magnetic spring based on the spacing between two magnets.

The repulsive force of the magnetic spring was measured according to the spacing between the magnets. As displayed in Figure 6, the repulsive force changed nonlinearly according to the spacing between the magnets: the smaller the spacing was, the more rapidly the repulsive force of the magnetic spring increased. Furthermore, as illustrated in Table 3, the difference in the repulsive force was large even when a small magnet was used. The repulsive force of the magnetic spring was over 100 N at a spacing of 3 mm in this study. The regression equation of the repulsive force is presented in Equation (2) according to the spacing between the magnets in this study (size: outer diameter, 25 mm; inner diameter, 10 mm; thickness, 7 mm):

(2) $$y=0.5053{\;x}^{2}-16.69\;x+154.1,{\;R}^{2}=0.9888,$$

where y is the repulsive force [N] and x is the spacing between the two magnets [mm].

In general, a metal spring exhibits a spring restoring force proportional to the displacement; thus, the stiffness has a constant value regardless of the displacement. However, a magnetic spring exhibits a repulsive force that varies rapidly and nonlinearly according to the magnetic spring spacing; thus, the stiffness of the magnet spring changes nonlinearly as well. As illustrated in Figure 6 and Equation (2), as the spacing between the magnets approached zero, the repulsive force continued to increase, and the stiffness of the magnetic spring increased as well.

In the second experiment of the magnetic repulsive force tester, both the maximum force, which corresponded to the maximum dynamic load, and the resonance frequency were measured according to changes in the weight of the mass body, which corresponded to the generating core in the VEH device. The magnetic repulsive force tester was installed on a vibrator, as illustrated in Figure 5. The sine sweep vibration test was performed with a vibration acceleration of 0.5 G in the frequency range of 25 to 70 Hz to measure the maximum force and its resonance frequency. The initial spacing between the magnets was set to 7 mm at 57.66 N, which was close to approximately 50% of the maximum repulsive force of the magnetic spring, 111.44 N (3 mm being the smallest spacing between two magnets within the vibrator capacity), as displayed in Table 3. This spacing between two magnets, 7 mm, corresponded to the moving displacement of the generating core of the magnetic spring VEH device. For reference, the moving displacement of the metal spring VEH device with a mechanical stopper was 4 mm. This is a design advantage of the magnetic spring that makes it possible to increase the moving displacement of the generating core without the durability problem of the VEH device. The initial weight of the mass body was set to the weight of the generating core of the metal spring VEH device, 300 g, and the weight of the mass body was varied in the tests, as illustrated in Table 4.

**Figure 7:**
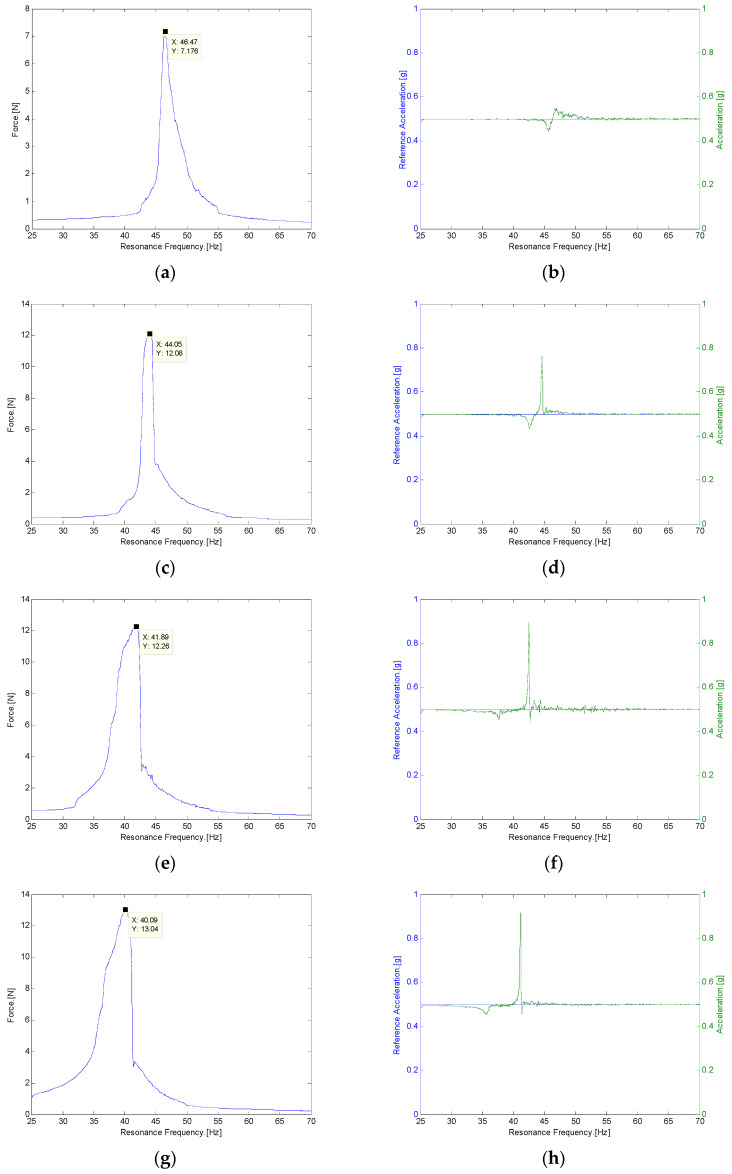
Maximum force and resonance frequency results according to changes in the weight of the mass body. (**Left side**) (**a**) Test 1: the maximum force was 7.176 N at a resonance frequency of 46.47 Hz with an input vibration acceleration of 0.5 G, where the weight of the mass body was 249.7 g (zoomed in); (**c**) Test 2: the maximum force was 12.08 N at a resonance frequency of 44.05 Hz with an input vibration acceleration of 0.5 G, where the weight of the mass body was 299.2 g; (**e**) Test 3: the maximum force was 12.26 N at a resonance frequency of 41.89 Hz with an input vibration acceleration of 0.5 G, where the weight of the mass body was 348.7 g; (**g**) Test 4: the maximum force was 13.04 N at a resonance frequency of 40.09 Hz with an input vibration acceleration of 0.5 G, where the weight of the mass body was 398.2 g; (**Right side**) (**b**,**d**,**f**,**h**) external vibration acceleration of 0.5 G (blue, reference) and the measured acceleration of the magnetic repulsive force tester (green) at the resonance frequency (zoomed out).

The maximum force was measured from 7.176 to 13.04 N at a vibration acceleration of 0.5 G, and the resonance frequency was varied from 46.47 to 40.09 Hz, as illustrated in Figure 7. Both the maximum force and resonance frequency were varied according to the weight of the mass body. Figure 7a,c,e,g represent the maximum force at the resonance frequency according to the weight of the mass body. In addition, Figure 7b,d,f,h compare the 0.5 G external vibration acceleration as a reference (in blue) and the actual measured vibration acceleration (in green) on the magnetic repulsive force tester at the resonance frequency according to the change in the weight of the mass body, as presented in Table 4. Compared with previous results, when using Equation (2), the repulsive force at a 1-mm spacing in the magnetic spring (137.92 N) was larger than 10 times the maximum force, which corresponded to the maximum dynamic load (13.04 N). Thus, the current magnet size in the magnetic spring was sufficient to support the change in the maximum dynamic load according to the change in the weight of the mass body from 250 to 400 g. Because the repulsive force of the magnetic spring changed nonlinearly with the spacing between the magnets, the smaller the spacing between the magnets was, the higher the repulsive force was.

Therefore, the magnetic spring VEH device can be applied at a location where a large shock vibration occurs during the driving of a high-speed train (i.e., axle box). The magnetic spring VEH device can support changes in the maximum dynamic load without damage and increase the moving displacement of the VEH device. In this study, the magnetic spring VEH device, which had the same generating core as the metal spring VEH device, was manufactured based on the test results, and its power generation performance was tested in the laboratory and in a high-speed train.

### 3.2. Laboratory Experiment to Test Power Generation Performance of Magnetic Spring VEH Device

**Figure 8:**
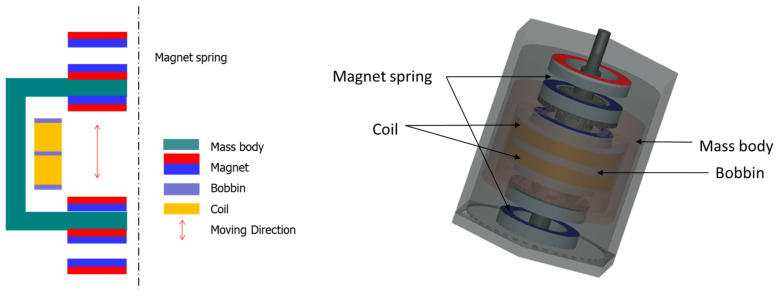
Schematic structure of magnetic spring vibration-energy-harvesting device.

Designing a VEH device with a smaller size and practical power generation performance is necessary to apply it to high-speed trains. To increase the generated power, a new design is needed to increase the magnetic field size, which is a significant factor in power generation performance when applied to high-speed trains. The design of the metal spring VEH device was researched in a previous study [5], and the basic design concept of the magnetic spring VEH device was based on the results, as illustrated in Figure 4 and Figure 8. The generating core of the magnetic spring VEH device used the same Nd magnets, which were arranged in pairs. Two coils were arranged similarly to correspond to each upper and lower magnet, as in the design of the metal spring VEH device, as illustrated in Figure 8.

The generating core was the moving component of the magnetic spring VEH device as the mass body and moved in the direction of motion. The weight of the generating core of the magnetic spring VEH device was 300 g ± 10 g, the same weight as in the metal spring VEH device and the magnetic repulsive force tester. Each coil of the magnetic spring VEH device could be connected in series or parallel. A total of 1000 turns of the coil at one coil bobbin (each lower and upper bobbin) were used in the generating core [2]. An internal resistance of 368 Ω was measured in one coil in the VEH device. The magnets in the magnetic spring were the same (ring-type permanent Nd magnets) and had the same size (outer diameter, 25 mm; inner diameter, 10 mm; thickness, 7 mm) as in the generating core of the metal spring VEH device. This was for ease of design and manufacture of the magnetic spring VHE device.

The magnetic spring was composed of a pair of Nd magnets at the top and bottom, as illustrated in Figure 8. The initial spacing between the two magnets corresponding to the moving displacement was set to 7 mm, as in the magnetic repulsive force tester. This was approximately 50% of the maximum repulsive force of the magnetic spring in the magnetic repulsive force tester within the vibrator capacity. The 7-mm spacing of the magnetic spring VEH device was greater than the 4-mm moving displacement of the metal spring VEH device with the mechanical stopper, which is an advantage of the magnetic spring. It is possible to accommodate an increase in moving displacement without durability problems in a magnetic spring. When the magnetic spring VHE device is applied to high-speed trains, it can improve the power generation due to the increase in the moving displacement of the generating core, as the increase implies receiving a large amount of energy from external vibrations and converting it into more incredible electrical energy. In addition, with large spacing between two magnets, it is expected that the generating core can move more easily even at a small external vibration acceleration during the driving of a high-speed train and generate more power.

**Figure 9:**
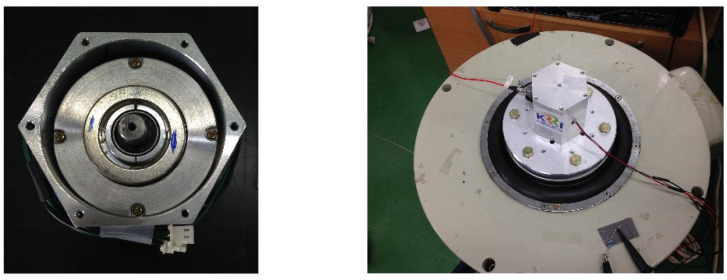
Magnetic spring vibration-energy-harvesting (VEH) device and magnetic spring VEH device on a vibrator in the laboratory.

Based on the test results of the maximum force and resonance frequency according to the weight of the generating core in Figure 7, the resonant frequency of the magnetic spring VEH device was estimated to be approximately 44 to 45 Hz when used with a generating core with a weight of 300 g ± 10 g, which was used in the metal spring VEH device. The schematic structure and pilot product of the magnetic spring VEH device in this study are illustrated in Figure 8 and Figure 9, respectively.

VEH devices use resonance. The closer the resonance frequency of the VEH device is to the frequency of the external vibration acceleration, the better the generation performance [2]. The resonance frequency of the VEH device is determined by the weight of the generating core and the stiffness of the spring [51]. In addition, the weight of the generating core does not change with vibration; therefore, if the stiffness of the spring is constant, the resonance frequency of the VEH device is also constant. However, as demonstrated in the previous magnetic repulsive force tester’s results, the stiffness of the magnetic spring changes nonlinearly according to the spacing of the magnet spring, which varies according to the external vibration acceleration. The resonance frequency also varies significantly and nonlinearly according to the external vibration acceleration, which results in a change in the power generation performance. Therefore, it is difficult to determine the power generation performance characteristics due to the nonlinearity of the spring stiffness of the magnetic spring VEH device. It is clear that the spring stiffness changes depending on the spacing of the magnetic spring, which corresponds to the moving displacement of the generating core; however, how these characteristics affect the power generation performance of the VEH device is unclear under various vibration conditions. Therefore, an experiment was conducted to examine how the nonlinear features of the magnetic spring affect the power generation performance of the magnetic spring VEH device for various vibration accelerations.

**Figure 10:**
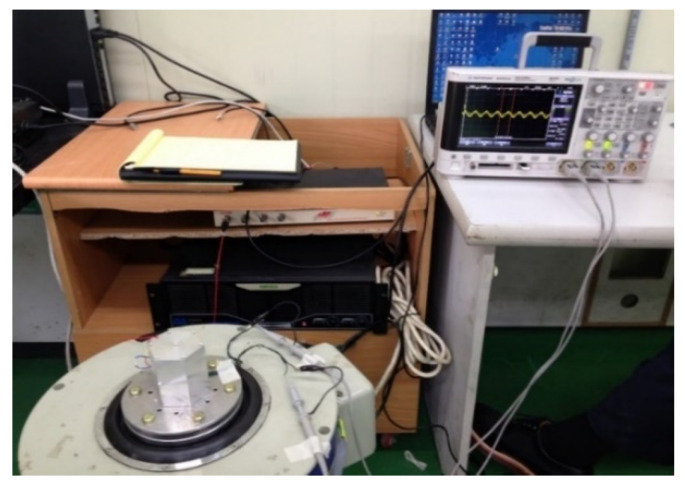
Performance test and measurement system for the magnetic spring vibration-energy-harvesting device in the laboratory.

The experiment to evaluate the resonance frequency and power generation performance of the magnetic spring VEH device was conducted under controlled laboratory conditions, as illustrated in Figure 10. The laboratory test system consisted of the following: a vibrator, a data recorder, and an accelerometer. The data acquisition board (DAQ) was used for the analog data, and MATLAB software was used to analyze the measured voltage data. The magnetic spring VEH device was tested with the frequency increased by 1 Hz/s linear forward sweeping excitation in the 20 to 70 Hz frequency band under vibration acceleration conditions of 0.5 G, 1.0 G, 1.5 G, and 2.0 G. The results were expressed for one coil in the VEH device for standardization of the test results, as in a previous study [2]. Each coil of the magnetic spring VEH device could be connected in series or in parallel. The maximum generated RMS voltage of the lower coil under the load condition (V*_rms-veh_*) was measured by connecting a 360-Ω load resistance, similar to the lower coil’s 368-Ω internal resistance in the lower coil of the magnetic spring VEH device. It was used for convenient testing using the load resistance. The experimental maximum generated power (P*_exp-veh_*) was calculated using Equation (1) with the maximum RMS value of the generated voltage (V*_rms__-veh_*) and the load resistance (R) [5,41]. The maximum RMS generated voltage (V*_rms__-veh_*) was used to calculate the average power the VEH device delivered to the load (i.e., the power dissipated across the load resistance).

**Figure 11:**
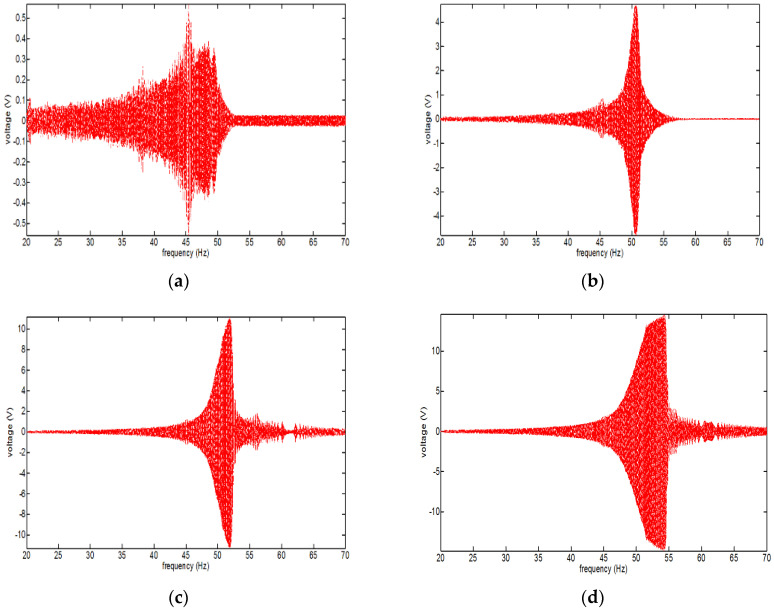
Performance test of the magnetic spring vibration-energy-harvesting device in the laboratory based on the magnitude of the external vibration acceleration: (**a**) 0.5 G vibration acceleration; (**b**) 1.0 G vibration acceleration; (**c**) 1.5 G vibration acceleration; (**d**) 2.0 G vibration acceleration.

The results of measuring the generated voltage according to the vibration acceleration are presented in Figure 11a–d. The resonance frequency range of the magnetic spring VEH device was predicted to be 44 to 45 Hz in the design stage. Figure 11a illustrates that the resonance frequency at the maximum generated voltage was 45.46 Hz at a vibration acceleration of 0.5 G, which was very close to the predicted resonance frequency in the design of the magnetic spring VEH device. However, as illustrated in Figure 11b–d, the resonance frequency at the maximum generated voltage was 50.57 Hz at a vibration acceleration of 1.0 G, 52.01 Hz at a vibration acceleration of 1.5 G, and 54.38 Hz at a vibration acceleration of 2.0 G. The range of resonance frequencies was thus 45.46 to 54.38 Hz at a vibration acceleration of 0.5 G to 2.0 G in this test.

In addition, the maximum generated power (P*_exp-veh_*) with a 360-Ω load resistance was calculated by the maximum RMS voltage (V*_rms-veh_*) and Equation (1) at each resonance frequency of the magnetic spring VEH device, as displayed in Table 5. The maximum RMS voltage (V*_rms-veh_*) was 0.56 to 14.58 V at a vibration acceleration of 0.5 G to 2.0 G, and the maximum values of P*_exp-veh_* were 0.87 to 590.48 mW at a vibration acceleration of 0.5 G to 2.0 G. However, increasing the maximum generated power (P*_exp-veh_*) of the magnetic spring VEH device was more significant than increasing V*_rms-veh_* while increasing the vibration acceleration. When the vibration acceleration changed from 0.5 G to 1.0 G, V*_rms-veh_* changed from 0.56 to 4.71 V, and P*_exp-veh_* changed from 0.87 to 61.62 mW. Furthermore, when the vibration acceleration changed from 1.5 G to 2.0 G, V*_rms-veh_* changed from 11.19 to 14.58 V, and P*_exp-veh_* changed from 347.82 to 590.48 mW, as indicated in Table 5.

However, as illustrated in Table 1 and Table 2, when comparing the maximum generated power (P*_exp-veh_*) of the metal spring VEH device with the mechanical stopper (moving displacement of 4.0 mm) to P*_exp-veh_* of the magnetic spring VEH device (moving displacement of 7.0 mm) under the same vibration acceleration conditions in the laboratory, P*_exp-veh_* of the magnetic spring VEH device was higher at a vibration acceleration of 1.5 G or higher than P*_exp-veh_* of the metal spring VEH device with the mechanical stopper. However, P*_exp-veh_* of the magnetic spring VEH device was lower at a vibration acceleration below 1.5 G. Furthermore, when compared with P*_exp-veh_* of the metal spring VEH device without a mechanical stopper (moving displacement of 8.0 mm), which did not consider durability, P*_exp-veh_* of the magnetic spring VEH device was lower in all vibration acceleration conditions, including vibration accelerations of 1.5 G and 2.0 G. These results differed from the expected power generation performance results with the exception of the durability improvement.

These results may have several causes. In the metal spring VEH device, the stiffness of the metal spring did not change, and power generation occurred continuously at the resonance frequency. However, the stiffness of the magnetic spring changed nonlinearly according to the spacing between magnets, which varied according to the vibration acceleration. The resonance frequency of the magnetic spring also varied significantly and nonlinearly according to the vibration acceleration; therefore, the resonance frequency of the magnetic spring VEH device was not consistent with the vibration acceleration. The motion of the generating core did not follow the frequency of the vibration acceleration. As a result, power generation did not occur continuously with vibration acceleration. In addition, when a small vibration acceleration is an input in the magnetic spring VEH device, the stiffness of the magnetic spring may be larger than the energy of the vibration acceleration; thus, the moving displacement of the generation core will be small, and the power generation performance will be degraded. In contrast, when a large vibration acceleration (1.5 G or higher in this study) is input, the energy of vibration acceleration may be larger than the stiffness of the magnetic spring; thus, the generating core will move sufficiently with the vibration acceleration, and the power generation performance will increase.

**Figure 12:**
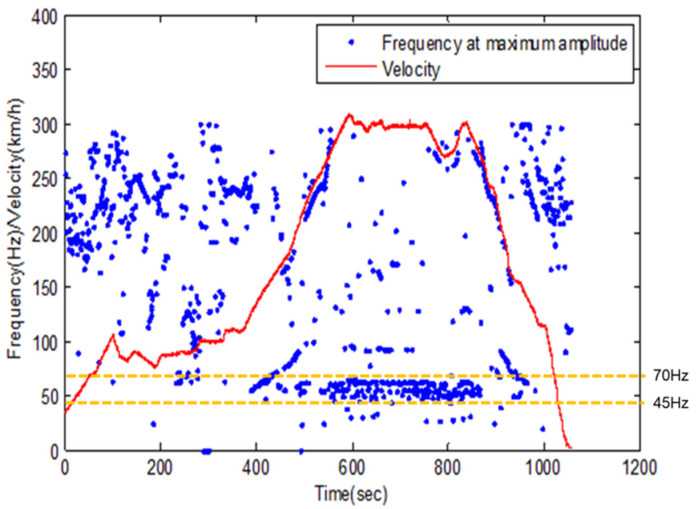
FFT results and velocity data. The resonance frequency occurred mainly in the frequency band between 45 and 70 Hz at 300 km/h, and the resonance frequency varied according to the train’s velocity.

Based on the test result of the power generation performance of the magnetic spring VEH device, it is difficult to predict which vibration conditions maximize the power generation performance of the magnetic spring VEH device. For example, as illustrated in Figure 12 [41], both the magnitude of vibration acceleration and the first main frequency constantly changed according to the train’s velocity on the axle box in the driving of the high-speed train by the previous study [41]; thus, it is difficult to predict how to change the resonance frequency of the magnetic spring VEH device to improve the power generation performance. Therefore, in the following experiment in this study, the magnetic spring VEH device and metal spring VEH device were installed in a high-speed train to compare and verify the power generation performance through an actual high-speed train test.

## 4. Experiment to Test Power Generation Performance of the Magnetic Spring VEH Device and Metal Spring VEH Device in a High-Speed Train

**Figure 13:**
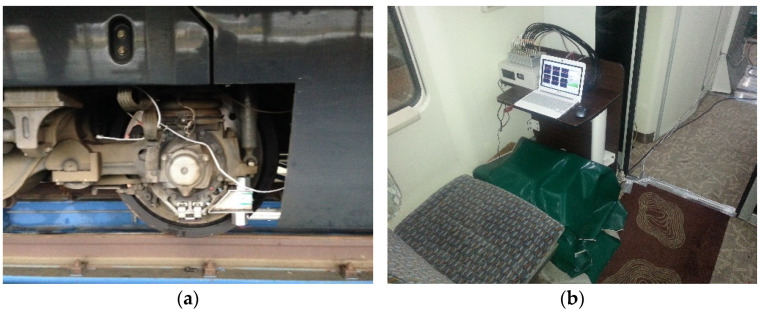
The VEH devices for the high-speed train test: (**a**) installation of two VEH devices on the axle box, where only the springs differed between the magnetic spring VEH device (bottom of the jig) and metal spring VEH device (top of the jig). The design of the resonance frequency of the two VEH devices was similar, approximately 45 Hz; (**b**) voltage measurement system in the cabin.

A number of experimental studies have been conducted on energy-harvesting power according to different design factors to verify the applicability of energy-harvesting technology that produces electrical energy from vibrational energy under testbed conditions [46,47,48]. A test of the power generation performance was conducted for the magnetic spring VEH device and metal spring VEH device with the mechanical stopper in a high-speed train. The magnetic spring VEH device and metal spring VEH device that were evaluated through laboratory tests were installed on the same axle box of a high-speed train to verify their performance simultaneously. The magnetic spring VEH device was installed on the bottom of the jig of the axle box of the high-speed train, and the metal spring VEH device was installed on the top of the jig, as illustrated in Figure 13a. Only the springs differed between the magnetic spring VEH device and metal spring VEH device. The design of the resonance frequency of the two VEH devices was similar, approximately 45 Hz. Each coil of the VEH devices and the voltage measurement system in the cabin were connected to measure the generated voltage in real-time during the high-speed train operation, as displayed in Figure 13b. The results were expressed for one coil in the VEH devices to standardize the test results. Each coil of the VEH devices could be connected in series or in parallel.

**Figure 14:**
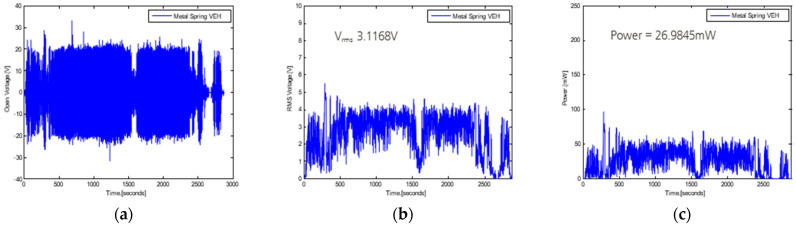
Performance test results of the metal vibration-energy-harvesting (VEH) device with a mechanical stopper, a 45-Hz resonance frequency, and a 360-Ω load resistance: (**a**) peak-to-peak voltage (V*_pk-pk-veh-T_*); (**b**) root mean square (RMS) generated voltages (V*_rms-veh-T_*); (**c**) experimentally generated power (P*_exp-veh-T_*).

**Figure 15:**
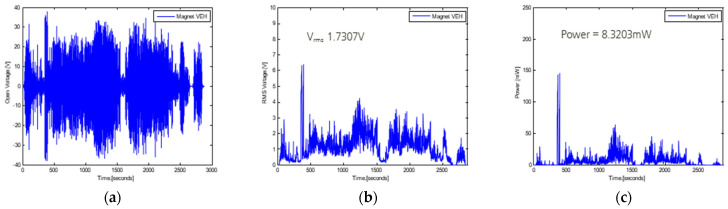
Performance test results of the magnet vibration-energy-harvesting (VEH) device, a 45-Hz resonance frequency, and a 360-Ω load resistance: (**a**) peak-to-peak voltage (V*_pk-pk-veh-T_*); (**b**) root mean square (RMS) generated voltages (V*_rms-veh-T_*); (**c**) experimentally generated power (P*_exp-veh-T_*).

The real-time generated voltages of both the magnetic spring VEH device and metal spring VEH device while the high-speed train was driving at approximately 300 km/h are presented in Figure 14 and Figure 15. The driving distance was 115.4 km (Busan station to East Daegu station in the southern area of Korea), and the driving test included a braking test of the high-speed train for approximately 1600 s, with slow driving of the train and stopping due to a train signal at approximately 2300 s.

The values of the peak-to-peak voltage generated with a 360-Ω load resistance in the high-speed train (V*_pk-pk-veh-T_*) are presented in Figure 14a and Figure 15a. The maximum values of V*_pk-pk-veh-T_* were very high, over ±30 V in both VEH devices. However, comparing the magnetic spring VEH device and metal spring VEH device, V*_pk-pk-veh-T_* of the magnetic spring VEH device rapidly changed from low to high voltage, by approximately ±30 V, during the change in driving speed of the high-speed train, as illustrated in Figure 15a. However, V*_pk-pk-veh-T_* of the metal spring VEH device was almost constant, approximately ±20 V, even with a change in the driving speed. This result was consistent with the difference in the moving displacement of the two VEH devices. The metal spring VEH device was limited to the moving displacement of the metal spring of 4 mm by the mechanical stopper. It could be observed that the voltage was relatively constant at approximately ±20 V in the high-speed train driving at a speed of 300 km/h. However, the moving displacement of the magnetic spring was 7 mm, and the stiffness of the magnetic spring changed nonlinearly according to the magnetic spring’s spacing, which changed according to the external vibration acceleration. For this reason, it could be observed that the voltage rapidly changed from low to high, by approximately ±30 V, in the high-speed train driving at a speed of 300 km/h.

The RMS voltages generated with a 360-Ω load resistance (V*_rms-veh-T_*) were calculated using a 1-s time interval of the peak-to-peak generated voltage with the load resistance at one lower coil. Figure 14b demonstrates that V*_rms-veh-T_* of the metal spring VEH device was 3.1168 V for the total time interval. In addition, the power of the metal spring VEH device generated experimentally in the high-speed train (P*_exp-veh-T_*) was 26.9845 mW for the same time interval by the results of V*_rms-veh-T_* and Equation (1), as illustrated in Figure 14c. Therefore, the total P*_exp-veh-T_* of the metal spring VEH device for the total harvested power output from the two coils (serial connection of the upper coil and lower coil) was 53.969 mW in the high-speed train test. This performance result was similar to that of a previous study [3], which demonstrated that the metal spring VEH device with a mechanical stopper met and exceeded the 50-mW threshold of the target power generation performance for the power source of a wireless sensor node in a high-speed train. However, the performance of the magnetic spring VEH device in the current study was very low, as illustrated in Figure 15b,c. The RMS voltage generated with a 360-Ω load resistance (V*_rms-veh-T_*) of the magnetic spring VEH device was 1.7307 V for the total time interval. In addition, the experimentally generated power (P*_exp-veh-T_*) of the magnetic spring VEH device was 8.3203 mW for the same time interval by V*_rms-veh-T_* and Equation (1). The value of the total P*_exp-veh-T_* of the magnetic spring VEH device for the total harvested power output from the two coils (serial connection) was as low as 16.6406 mW in the high-speed train test. Therefore, the magnetic spring VEH device did not meet the 50-mW threshold for the target power generation performance for the power source of a wireless sensor node in the train.

The difference in the total P*_exp-veh-T_* between the metal spring VEH device and magnetic spring VEH device was approximately 70%. Particularly in the case of the magnetic spring VEH device, the stiffness of the magnetic spring changed nonlinearly according to the magnet spring’s spacing, which varied according to the external vibration acceleration. The resonance frequency also varied significantly and nonlinearly according to the external vibration acceleration of the driving speed. The maximum generated power (P*_exp-veh-T_*) was very high, approximately 150 mW at 1 s, by the high vibration acceleration, and it was larger than P*_exp-veh-T_* of the metal spring VEH device. However, the average generated power of the magnetic spring VEH device was smaller than that of the metal spring VEH device.

Table 2 and Table 5 can be used to identify the cause of the train’s test results. The metal spring VHE device can generate power even at a low vibration acceleration if the resonance frequency matches that of the metal spring VEH device. However, in the magnetic spring VEH device, the stiffness of both magnet springs and the resonance frequency change according to the vibration acceleration during high-speed train driving. The generated power (P*_exp-veh-T_*) of the magnetic spring VEH device thus changes continuously. When a small vibration acceleration is an input in the magnetic spring VEH device, the stiffness of the magnetic spring may be larger than the energy of vibration acceleration; therefore, the moving displacement of the generation core will be small, and the power generation performance will be degraded. In contrast, when a large vibration acceleration is an input, the energy of vibration acceleration may be larger than the stiffness of the magnetic spring; thus, the generating core will move sufficiently with the vibration acceleration, and the power generation performance will increase.

The power generated by a general VEH device depends on the weight of the generating core, the moving displacement, the stiffness of the magnetic spring, and the resonance frequency according to the vibration acceleration. To meet or exceed 50 mW for the target power generation needed for a high-speed train, the weight of the generating core in the current magnetic spring VEH device should be heavier or the moving displacement should be increased. In addition, the nonlinearity of the stiffness of the magnetic spring due to the vibration acceleration needs to be addressed. Therefore, changing only one of the design factors of the magnetic spring VEH device is likely insufficient to meet the target power generation performance.

Further research on the magnetic spring VEH device will be conducted in future work. However, according to the current study, the magnetic spring VEH device results differed from the expected results in improving the power generation performance, with the exception of the durability improvement in the laboratory experiment and high-speed train test. Therefore, only the metal spring VEH device with a mechanical stopper meets the durability and target power generation requirements for use as a power source for wireless sensor nodes in high-speed trains.

## 5. Results

Durability is one of the most critical problems in the design of a VEH device. Despite the VEH device’s excellent performance, moving components, such as the metal spring, were damaged during the operation of a high-speed train. Therefore, to solve the durability problem of the metal spring in the VEH device, this study applied a non-contact magnetic spring using the repulsive force of a permanent magnet to the VEH device, and conducted a laboratory experiment and high-speed train experiment as a preliminary study.

In the laboratory experiment, the repulsive force varied nonlinearly according to the spacing between the magnets, and the difference in the repulsive force was enormous even when a small magnet was used. The repulsive force of the magnetic spring was over 100 N at a spacing of 3 mm in this study. In the second experiment, the maximum force was measured from 7.176 to 13.04 N at a vibration acceleration of 0.5 G, and the resonance frequency also varied from 46.47 to 40.09 Hz. Both the maximum force and the resonance frequency changed according to the weight of the mass body. Comparing the results, the repulsive force at a 1-mm spacing in magnetic spring (137.92 N) was larger than 10 times the maximum force, which corresponded to the maximum dynamic load (13.04 N). Therefore, the use of the current magnet size in the magnetic spring can sufficiently support changes in the maximum dynamic load according to the change in the weights of the mass body in the range of 250 to 400 g. An experiment was also conducted to determine the properties of the magnetic spring. A magnetic spring VEH device was designed and manufactured to test the power generation performance in the laboratory and in a high-speed train. The resonance frequency range of the magnetic spring VEH device was predicted to be 44 to 45 Hz in the design stage. The resonance frequency at the maximum generated voltage was 45.46 Hz at a vibration acceleration of 0.5 G, which was very close to the predicted resonance frequency in the design of the magnetic spring VEH device. However, the resonance frequency at the maximum generated voltage was 50.57 Hz at a vibration acceleration of 1.0 G, 52.01 Hz at a vibration acceleration of 1.5 G, and 54.38 Hz at a vibration acceleration of 2.0 G. The range of resonance frequencies was thus 45.46 to 54.38 Hz at a vibration acceleration of 0.5 G to 2.0 G in this test. In addition, the maximum generated power (P*_exp-veh_*) with a 360-Ω load resistance was calculated by the maximum RMS voltage (V*_rms-veh_*) and Equation (1) at each resonance frequency of the magnetic spring VEH device. Increasing the maximum generated power (P*_exp-veh_*) of the magnetic spring VEH device was more significant than increasing V*_rms-veh_* while increasing the vibration acceleration.

Furthermore, when P*_exp-veh_* of the metal spring VEH device with a mechanical stopper was compared with P*_exp-veh_* of the magnetic spring VEH device under the same vibration acceleration conditions in the laboratory, P*_exp-veh_* of the magnetic spring VEH device was higher at a vibration acceleration of 1.5 G or higher than P*_exp-veh_* of the metal spring VEH device with a mechanical stopper. However, P*_exp-veh_* of the magnetic spring VEH device was lower at a vibration acceleration below 1.5 G. Furthermore, when compared to the metal spring VEH device without a mechanical stopper, which did not consider durability, P*_exp-veh_* of the magnetic spring VEH device was lower in all vibration acceleration conditions, including a vibration acceleration of 1.5 G and 2.0 G. These results were not consistent with the expected power generation performance improvement except for the durability improvement. 

Finally, a test of the power generation performance was conducted for the magnetic spring VEH device and metal spring VEH device with the mechanical stopper in a high-speed train. The total P*_exp-veh-T_* of the metal spring VEH device for the total harvested power output from the two coils of the metal spring VEH device was 53.969 mW in the high-speed train test. In contrast, the performance of the magnetic spring VEH device was very low. The total P*_exp-veh-T_* of the magnetic spring VEH device for the total harvested power output from the two coils (serial connection) was as low as 16.6406 mW in the high-speed train test. The difference in the total P*_exp-veh-T_* between the metal spring VEH device and magnetic spring VEH device was approximately 70%. 

The magnetic spring VEH device must meet or exceed 50 mW for the target power generation needed for a high-speed train. To meet this requirement, in the current design of the magnetic spring VEH device, the weight of the generating core would be heavier or the moving displacement would be increased. In addition, the nonlinearity of the stiffness of the magnetic spring based on the vibration acceleration would have to be addressed. Therefore, changing only one of the design factors in the magnetic spring VEH device is insufficient to meet the target power generation performance.

## Figures and Tables

**Table 1:** Maximum generated power with a 360-Ω load resistance (P*_exp-veh_*) at one lower coil of the metal vibration-energy-harvesting device without the mechanical stopper. The maximum moving displacement of the spring was 8.0 mm (±4.0 mm).

Vibration Acceleration (G)	Vibration Displacement (mm)	Maximum RMS Voltage, V*_rms-veh_* (V)	Maximum Generated Power, P*_exp-veh_* (mW)
0.5	2.18	9.78	265.69
1.0	3.25	15.11	634.20
2.0	3.81	16.60	765.44

**Table 2:** Maximum generated power with a 360-Ω load resistance (P*_exp-veh_*) at one lower coil of the metal vibration-energy-harvesting device with the mechanical stopper. The maximum moving displacement of the spring was 4.0 mm (±2.0 mm).

Vibration Acceleration (G)	Vibration Displacement (mm)	Maximum RMS Voltage, V*_rms-veh_* (V)	Maximum Generated Power, P*_exp-veh_* (mW)
0.5	1.88	8.01	178.22
1.0	2.05	8.53	202.11
2.0	2.21	9.02	226.00
3.0	2.34	9.41	245.97

**Table 3:** Measurement results of the repulsive force [N] at the gap between two magnets [mm].

Spacing of Two Magnets (mm)	3	4	6	7	9	10	12	13
Repulsive Force (N)	114.44	95.05	67.31	57.66	43.07	35.84	29.64	25.36

**Table 4:** Weight of mass body in different test conditions for the magnetic repulsive force tester.

The Number of the Test	1	2	3	4
The weight of the mass body (g)	249.7	299.2	348.7	398.2

**Table 5:** Maximum generated power with a 360-Ω load resistance (P*_exp-veh_*) at one lower coil of the magnetic spring vibration-energy-harvesting device. The maximum moving displacement of the spring was 7 mm.

Vibration Acceleration (G)	Resonance Frequency (Hz)	Maximum RMS Voltage, V*_rms-veh_* (V)	Maximum Generated Power, P*_exp-veh_* (mW)
0.5	45.46	0.56	0.87
1.0	50.57	4.71	61.62
1.5	52.01	11.19	347.82
2.0	54.38	14.58	590.48
